# Fear of COVID-19, Stress, Fear of Childbirth, Hardiness and Life Satisfaction in Polish Pregnant Women During the Pandemic

**DOI:** 10.3390/jcm14196842

**Published:** 2025-09-26

**Authors:** Joanna Dymecka, Kinga Marszałek-Mucha, Anna Machnik-Czerwik

**Affiliations:** 1 Department of Health Psychology and Quality of Life, Institute of Psychology, Opole University, 45-052 Opole, Poland; jdymecka@uni.opole.pl; 2 Faculty of Social Sciences, Institute of Psychology, Opole University, 45-052 Opole, Poland

**Keywords:** hardiness, life satisfaction, fear of childbirth

## Abstract

**Introduction:** Pregnancy is a period of many challenges and changes that women face. These challenges include being pregnant during the COVID-19 pandemic, as well as preparing for childbirth. Personal resources, such as hardiness, may help to adapt to these difficult life events. **Objective:** The aim of this study was to determine the relationship between fear of COVID-19, stress, fear of childbirth, hardiness, and life satisfaction in pregnant women and identify two predictors, fear of childbirth and life satisfaction, in pregnant women during the pandemic. **Method:** This study involved 247 pregnant women aged 18 to 39. Five tools were used: the Labour Anxiety Questionnaire (KLP II), the Health-Related Hardiness Scale (HRHS), the Pandemic-Related Pregnancy Stress Scale (PREPS), the Satisfaction with Life Scale (SWLS), and the Fear of COVID-19 Questionnaire (FOC-6). **Results:** Significant positive correlations were observed between fear of COVID-19 and perinatal infection stress (r = 0.74, *p* < 0.001), preparedness stress (r = 0.51, *p* < 0.001), and fear of childbirth (r = 0.32, *p* < 0.001). Perinatal infection stress was positively associated with preparedness stress (r = 0.53, *p* < 0.001) and fear of childbirth (r = 0.33, *p* < 0.001). Preparedness stress was positively correlated with fear of childbirth (r = 0.47, *p* < 0.001). Fear of childbirth was negatively correlated with hardiness (r = −0.22, *p* < 0.001) and life satisfaction (r = −0.29, *p* < 0.001). The predictors of fear of childbirth are preparedness stress associated with the organization of childbirth (β = 0.38, t = 5.60, *p* < 0.001) and hardiness (β = −0.16, t = −2.84, *p* = 0.00, *p* < 0.01), while the predictor of satisfaction with life is fear of childbirth (β = −0.31, t = −4.28, *p* = 0.00, *p* < 0.001). **Conclusions:** Epidemics of infectious diseases may have a negative emotional impact on pregnant women, causing fear and stress and contributing to an increased level of fear of childbirth, which can lead to complications for the health of the mother and child. Therefore, it is particularly important to support women preparing for childbirth during subsequent pandemics or other crisis situations. Personal resources, such as hardiness, are important for experiencing fear of childbirth, which is why pregnant women who experience increased fear and stress should have their resources strengthened.

## 1. Introduction

COVID-19 (Coronavirus Disease 2019), the infectious disease caused by SARS-CoV-2 (Severe Acute Respiratory Syndrome Coronavirus 2), posed a serious threat to the health and lives of people around the world. Data on the course of infection in pregnant women were contradictory and ambiguous. Pregnant women were initially considered a risk group for severe COVID-19 due to physiological and immunological changes that make pregnant women more susceptible to infections [1,2,3]. Subsequently, it was found that the clinical symptoms of SARS-CoV-2 infection are the same as in non-pregnant patients [4]. Pregnant women were more likely to be hospitalized, more likely to require oxygen support, more likely to be admitted to intensive care units, and had higher mortality [5]. Due to the fact that pregnant women during the pandemic were at increased risk, they had numerous concerns about their own and their child’s health and life [6]. They were also worried about the functioning of the healthcare system during the pandemic, such as limited access to doctors or closing of hospital wards [7]. Two main sources of stress are indicated. The first source is the fear of infection, and the second is the organization of childbirth during the pandemic. Both types of stress are associated with increased anxiety. About one third of pregnant women during the pandemic experienced stress, both related to preparation for childbirth and the risk of infection [8,9]. Increased stress during pregnancy may contribute to increased fear of childbirth (FOC).

Childbirth is one of the most extreme experiences in a woman’s life [10]. Although it is certainly a long-awaited moment of meeting the child, it arouses very strong fear in women. Fear of childbirth (FOC) is conceptually distinct from generalized pregnancy anxiety, as it specifically refers to worries and apprehensions related to the process of labor and delivery, including pain, complications, and loss of control during birth, whereas generalized pregnancy anxiety encompasses broader concerns about the health and well-being of both the mother and child throughout pregnancy. Fears related to childbirth are the result of social learning. Important sources of fear are the reports of other women, messages available in the mass media, and information obtained from the Internet. Before childbirth, women feel fear primarily of severe pain. Fear of childbirth is also associated with a threat to the health of the child and the mother, the possibility of complications, giving birth to a child with a defect, hospitalization, obstetric violence, inappropriate behavior during childbirth, and lack of control over one’s own behavior, especially screaming [11,12]. Fear of childbirth during the pandemic may also be related to the lack of family births and support, hospital isolation, and COVID-19 infection, which may affect the type of delivery, its course, and the approach of the staff [7].

All of this may affect the well-being and life satisfaction of pregnant women during the pandemic. According to Veenhoven [13], life satisfaction is one of the indicators of quality of life. Together with mental and physical health, it indicates how well people function. In his opinion, life satisfaction is the degree to which a person positively assesses the overall quality of their entire life. According to Diener [14], subjective well-being is the broadest concept in this scope and can be understood as a multi-dimensional concept of both affective and cognitive nature. In his opinion, life satisfaction is a component of well-being and, according to him, it is a construct representing a cognitive and global assessment of the quality of life as a whole. Therefore, life satisfaction in his approach is an assessment, a component of subjective well-being, which is partly independent and partly related to the affective aspects of well-being [15]. The abovementioned concept shows that life satisfaction can be influenced by experienced emotions and stress. The COVID-19 pandemic has brought about many negative emotions and an increase in the level of stress in society [16]. People experience fear of infection, illness, complications, or the death of a loved one. However, as research indicates, not all people will experience the negative consequences of the pandemic in the same way. Among the factors that positively influence people’s life satisfaction during the COVID-19 pandemic, we can mention, among others, personal resources such as the sense of coherence [17].

Drawing on Antonovsky’s Salutogenic Theory and the sense of coherence (SOC) framework, hardiness is regarded as a personal resource that enables individuals to cope effectively with stress and support their overall well-being. Hardiness is one of the resources whose importance is analyzed in the context of protecting an individual from the negative impact of the COVID-19 pandemic. Hardiness is usually defined as a generalized style of functioning of an individual characterized by a high level of commitment, control, and challenge, thanks to which the negative effects of stress are mitigated. It determines how an individual approaches events and how they interpret them. People with a high level of hardiness believe that they have control over their lives and that by engaging in set goals, they will achieve positive results, and they treat everyday stressors as challenges. The concept of hardiness is based on the assumption that stressful circumstances are an inherent part of life and that coping with them effectively is necessary to be able to grow and develop [18]. Hardiness can improve an individual’s well-being and increase their life satisfaction. Many studies have been conducted in different populations, which have shown that hardiness is positively associated with quality of life and life satisfaction [19,20,21]. Several studies conducted during the COVID-19 pandemic have shown that hardiness is a very important resource in coping with adverse events resulting from COVID-19 [22,23]. It is an important resource in coping with the challenges of the COVID-19 pandemic and a mediator of the relationship between fear of COVID-19 and life satisfaction [17].

Therefore, it can be assumed that hardiness may be an important resource for pregnant women to cope with the negative effects of the COVID-19 pandemic and a significant predictor of fear of childbirth and life satisfaction. The aim of the current study was to analyze the relationship between fear of COVID-19, stress experienced by pregnant women, fear of childbirth, and hardiness and life satisfaction and to determine the predictors of fear of childbirth and life satisfaction.

In line with the theoretical background and previous findings presented above, the study addressed the following research questions and tested the following hypotheses:

1. What are the relationships between fear of COVID-19, perinatal infection stress, preparedness stress, fear of childbirth, hardiness, and life satisfaction in pregnant women during the COVID-19 pandemic?
2. Do sociodemographic variables related to the course of the pandemic, the functioning of the healthcare system, and the health status of women differentiate the studied group in terms of the level of fear of COVID-19, stress, fear of childbirth, mental toughness, and life satisfaction?
3. Which factors predict fear of childbirth in pregnant women?
4. Which factors predict life satisfaction in pregnant women during the COVID-19 pandemic?

Hypotheses

**H1.**  *Fear of COVID-19 will be positively correlated with perinatal infection stress, preparedness stress, and fear of childbirth. Perinatal infection stress and preparedness stress will be positively correlated with fear of childbirth. Fear of childbirth will be negatively correlated with hardiness and life satisfaction*.

**H2.**  *Sociodemographic variables (e.g., age, access to family birth, previous COVID-19 infection) differentiate the levels of the studied variables*.

**H3.**  *Preparedness stress and hardiness will be significant predictors of fear of childbirth*.

**H4.**  *Fear of childbirth and hardiness will be a significant predictor of life satisfaction*.

## 2. Materials and Methods

### 2.1. Subjects and Procedure

This study was conducted among 247 pregnant women in 2021. Due to the epidemiological situation, the study was conducted online. The study participants were informed about the purpose of the study, that the study was anonymous, and that the results would only be used for scientific purposes. The study was not time-limited. All participants agreed to participate in the study. The study participants could withdraw from the study at any time during its duration. The study received the consent of the Ethical Committee of the University of Opole (KEBN 15/2021).

### 2.2. Measures

The following research tools were used in the study: Labour Anxiety Questionnaire (KLP II), The Health-Related Hardiness Scale (HRHS), The Pandemic-Related Pregnancy Stress Scale (PREPS), the Satisfaction with Life Scale (SWLS), and the Fear of COVID-19 Questionnaire (FOC-6).

Fear of childbirth was measured by the Labour Anxiety Questionnaire (KLP II), which consists of nine statements. Respondents answer the questions using a 4-point scale, from “definitely not” to “definitely yes”. The result ranges from 0 to 27 points. The higher the score, the higher the intensity of fear of childbirth. The validity and reliability of the questionnaire are high and satisfactory [24].

Hardiness was measured by the Health-Related Hardiness Scale (HRHS) by Pollock in the Polish adaptation by Dymecka et al. [19,25]. The scale was developed to measure hardiness related to health problems. The scale consists of twelve statements. For each item, respondents answer using a 6-point scale, from “strongly disagree” to “strongly agree”. The reliability of the scale is 0.89. The higher the score, the higher the level of hardiness.

Stress was measured by the Pandemic-Related Pregnancy Stress Scale (PREPS), by Preis, Mahaffey & Lobel in the Polish adaptation by Ilska et al. [8,9]. The scale consists of 17 statements. Respondents answer to each item using a 5-point scale from “very little” to “very much”: the questionnaire consists of three subscales: Perinatal Infection Stress (5 items), Preparedness Stress (7 items), and Positive Appraisal (3 items). The scale is characterized by acceptable reliability (Cronbach’s α = 0.86).

Life satisfaction was measured by The Satisfaction with Life Scale by Diener et al. in the Polish version by Czapiński [14,26]. The scale consists of five statements. The participants respond to each item using a scale from 1 to 7. The reliability of the scale is acceptable (α = 0.87). The higher the score, the greater the satisfaction with life.

Polish Fear of COVID-19 Scale (FOC-6) by Dymecka et al. [16] was used to measure fear of the coronavirus. The scale consist of 6 statements regarding subjective thoughts and feelings experienced in the last month in connection with the ongoing COVID-19 pandemic. Respondents answer each item on a 5-point scale from “strongly disagree” to “strongly agree”. The reliability index of the scale is 0.83.

We performed a survey of demographic and pregnancy-related variables, which consisted of two parts. The first part contained questions on sociodemographic data, such as age or education. The second part contained questions on pregnancy and health status, such as number of pregnancies, chronic diseases, complications, perinatal problems, and hospitalizations.

### 2.3. Statistical Analysis

Descriptive statistics were analyzed using the Shapiro–Wilk test to check whether the distribution of variables differed from normal. Parametric tests were used in the analysis. Pearson’s r correlation was used to examine the relationships between variables. Student’s *t*-test for independent samples was used to check differences in the level of the analyzed variables between the two groups. Multiple linear regression analyses were performed for the dependent variables, fear of childbirth and life satisfaction. All statistical analyses were conducted using Statistica software, version 13. The sample size (N = 247) was sufficient to achieve adequate statistical power for the analyses performed (correlations, regression, *t*-tests).

## 3. Results

In total, 247 pregnant women participated in the study, aged 18 to 39 years (M = 27.17, SD = 4.21). Most of the examined women had higher education (n = 150; 60.73%), about 1/3 were women who had undergone secondary education (n = 82; 33.20%), 12 women had vocational education (n = 12; 4.86%), and 3 women had only completed primary school (1.21%). The studied population varied in terms of the stage of pregnancy. The study included women from the 5th to the 40th week of pregnancy (M = 26.38, SD = 8.78). Most of the examined women did not suffer from chronic diseases (75.30%). The participants’ sociodemographic data are presented in Table 1.

Descriptive statistical analyses and the Shapiro–Wilk test were performed for all variables. The results showed that the distribution of most variables was different from normal (see Table 2). Although the Shapiro–Wilk test indicated significant deviations from normality (*p* < 0.001), the use of parametric tests in this study is justified due to the sufficiently large sample size (N = 247). Research has shown that tests such as the independent-samples *t*-test and linear regression are robust to violations of normality in large samples, allowing for reliable inference despite non-normal distributions [27,28]. The analysis of skewness and kurtosis also showed that all tested variables did not show significant asymmetry. Taking into account the slight asymmetry of the tested variables and the size of the sample, we decided to use parametric analyses to verify the hypotheses [29].

In order to determine whether there is a relationship between fear of COVID-19, stress, fear of childbirth, hardiness, and life satisfaction in the studied group of pregnant women, Pearson’s r correlation was performed. The obtained results are shown in Table 3.

The analysis showed that there is a positive relationship between fear of COVID-19 and perinatal infection stress, preparedness stress, positive appraisal, and fear of childbirth. The strength of these relationships is from moderate to strong (correlation coefficients range from 0.22 to 0.74). Preparedness stress and fear of childbirth are weakly and negatively correlated with hardiness. The higher the level of preparedness stress and fear of childbirth, the lower the level of hardiness. Positive appraisal is additionally positively associated with life satisfaction. Fear of childbirth is negatively correlated with hardiness and life satisfaction. In addition, there is a positive relationship between hardiness and life satisfaction. In order to check whether there is a relationship between the age of the pregnant women, the number of pregnancies and deliveries, and the stage of pregnancy and the level of psychological variables, Pearson’s r correlation was used. The results are presented in Table 4.

There are weak positive relationships between the age of the respondents and fear of COVID-19, hardiness, and life satisfaction. Age is negatively correlated with preparedness stress. The number of pregnancies and deliveries negatively correlates with preparedness stress and fear of childbirth. This suggests that the greater the number of pregnancies and deliveries, the lower the perceived stress and fear of childbirth. In addition, the week of pregnancy is positively associated with hardiness.

Additionally, we checked whether there were differences in the level of the analyzed variables between women planning a family childbirth and those who did not plan such a childbirth. Student’s parametric *t*-test for independent samples was used (see Table 5).

It has been shown that there are significant differences in the level of preparedness stress and fear of childbirth between women planning a family birth and those who do not plan such a birth. Women planning a family birth during the pandemic experience greater stress and fear of childbirth than women who do not plan this type of birth.

### Predictors of Fear of Childbirth and Satisfaction with Life

The next step of the analysis was to check whether fear of COVID-19, stress, and hardiness are predictors of fear of childbirth and whether the above variables together with fear of childbirth are predictors of satisfaction with life. For this purpose, regression analysis was used. Data are presented in Table 6 and Table 7. The level of fear of childbirth is determined by preparedness stress (Beta = 0.38, t = 5.60, *p* < 0.001) and hardiness (Beta = −0.16, t = −2.84, *p* = 0.00, *p* < 0.01). The regression model is significant (F = 20.8, *p* < 0.001) and explains 24% of the variance of the fear of childbirth variable.

The regression model predicting fear of childbirth was statistically significant (R^2^ = 0.24, F(4,242) = 20.8, *p* < 0.001), indicating a medium-to-large overall effect (Cohen’s f^2^ = 0.32). Examination of the individual predictors showed that preparedness stress had a medium effect (f^2^ ≈ 0.16) and hardiness a small effect (f^2^ ≈ 0.03). These results suggest that although all predictors contribute to the model, preparedness stress is the most influential factor in predicting fear of childbirth.

The above data suggest that the level of life satisfaction is determined by the fear of childbirth (Beta = −0.31, t = −4.28, *p* = 0.00, *p* < 0.001). The regression model is significant (F = 6.13, *p* < 0.05) and explains 13% of the variance of the life satisfaction variable.

## 4. Discussion

The aim of this study was to analyze the relationship between stress and fear experienced during the COVID-19 pandemic and the fear of childbirth, hardiness, and life satisfaction of pregnant women. Every pregnancy is associated with increased stress [30]. Difficult situations, such as the COVID-19 pandemic, intensify this stress [7]. In this study, we showed that there is a relationship between fear of COVID-19 and stress in pregnant women, both related to the risk of infection and changes in the organization of childbirth and obstetric care. Other studies have shown that fear of COVID-19 negatively affects an individual’s mental health and significantly increases the level of psychological stress [16,31,32]. Fear of COVID-19 is positively associated with negative emotions, and concerns about the risk of infection increase anxiety and stress [33]. Studies show that pregnant women experience stress both due to risk of infection and complications during pregnancy, as well as due to changes in the course of labor and related restrictions [8].

The analysis also showed a significant relationship between fear of COVID-19 and pandemic stress and fear of childbirth. Childbirth is associated with a hospital stay, contact with other people, and an increased risk of infection. In addition, changes in perinatal care mean that women are often forced to give birth in a mask, have a cesarean section, or are separated from their newborn. In addition, family births and hospital visits have been limited or completely banned during the pandemic. Therefore, pregnant women who feel fear of COVID-19 also feel fear of childbirth. These results were also confirmed in our other studies [7,34].

There was no relationship between fear of COVID-19, stress, and the level of life satisfaction, which is inconsistent with the results of other studies, which indicate that fear of COVID-19 and stress are negatively associated with life satisfaction [16,35]. Studies conducted in Turkey also showed that fear of COVID-19 is negatively associated with life satisfaction and positively correlated with anxiety and stress. It was also confirmed that fear of COVID-19 increases stress and anxiety and reduces life satisfaction [32]. Most likely, in the case of pregnant women, other variables, such as fear of childbirth, social support, family relationships, or complications during pregnancy, are more associated with life satisfaction than the fear of COVID-19 infection itself.

However, a relationship between life satisfaction and fear of childbirth, positive appraisal, and hardiness has been demonstrated. Childbirth is a difficult situation, especially during a pandemic. The prevailing conditions generate substantial uncertainty for women concerning expectations during pregnancy and childbirth. The threat is the virus itself, as well as many serious consequences and complications that have caused restrictions and limitations. Additionally, the restriction associated with family deliveries, the ban on visits, and uncertainty about the test result and the situation in the hospital may increase the level of fear of childbirth and stress in the future mother [7,36]. It may be related to the woman’s daily functioning and her satisfaction with life. This study showed a weak negative correlation between stress related to the organization of childbirth and fear of childbirth and hardiness. Other studies have also shown that people with a high level of hardiness show lower levels of perceived stress [37]. Hardiness is a resource responsible for the way an individual can efficiently cope with emerging challenges and problems of everyday life as well as pressure, stress, anxiety, and negative emotions [38]. In the current epidemiological situation, fear of childbirth in a group of pregnant women does not only concern the course of childbirth and labor pain but also childbirth without the support of a partner, possible infection, isolation, and many negative health consequences for the mother and the child. Studies have shown that hardiness is an important resource in coping with the pandemic situation, including negative emotions, fear, stress, and anxiety. Hardiness is an adaptive resource in stressful situations related to the COVID-19 pandemic. People with a high level of this trait interpret stressful life events as less burdensome. A high level of hardiness can help a person control anxiety, stress, and fear [39]. A weak positive correlation has been shown between hardiness and life satisfaction. Other studies also show that people with a high level of hardiness have a higher quality of life and are more optimistic [40]. Hardiness prevents the deterioration of psychological well-being in difficult situations [41,42]. Studies indicate that hardiness is positively associated with life satisfaction during the COVID-19 pandemic [39]. Other studies also show that hardiness is positively associated with quality of life [20,43,44].

In this study, we also showed that women’s age is weakly and positively correlated with fear of COVID-19. The older the woman, the more serious the course of COVID-19 [16], and it is also more difficult to get pregnant, more difficult to maintain it, and the risk for both the mother and the child is greater [45]. Late pregnancy may be associated with more complications or comorbidities such as hypertension or diabetes. The results of previous studies indicate a frequent occurrence of pregnancy complications in older women [46]. Pregnancy at a later age is also associated with a higher risk of hospitalization [46,47]. Hospitalization itself may be associated with a higher risk of coronavirus infection. In addition, women who become pregnant at a later age often previously experienced gynecological and obstetric complications, infertility, or miscarriages. Therefore, as the literature shows, they may be accompanied by stress, anxiety, and fear [48,49]. It can be assumed that such women also experience a greater level of fear of COVID-19. In addition, a weak negative correlation was shown between age and stress related to the organization of deliveries. The simultaneous negative correlation with the number of pregnancies may indicate the fact that older women who are pregnant again are more aware of what awaits them and have knowledge about the course of delivery. A study among young non-pregnant women confirms that the thought of a possible pregnancy in the future arouses stress and anxiety [50]. Age was positively correlated with hardiness and life satisfaction. According to Antonovsky’s concept [51], the level of personal resources is established after the age of 30. Therefore, older people are characterized by a higher level of resources compared to teenagers or young adults. The situation is similar in the case of life satisfaction. Younger people with a lower level of personal resources and with lower self-esteem are characterized by lower life satisfaction. The COVID-19 pandemic has caused changes in the organization of the functioning of healthcare, including changes in the organization of the course of labor, especially during the intensification of waves and an increased frequency of illnesses, among others, meaning that it was impossible to have family births. The current study analyzed whether planning a family birth was a variable differentiating the level of the variables studied. It has been shown that women who planned a family birth experience greater stress related to the organization of the birth and greater fear of childbirth. In our study, over 74% of women declared that they planned a family birth. In another Polish study, over 85% of women declared that they wanted to give birth in the presence of a companion. Pregnant women most often motivated their decision with psychological support, the company of a close person, and ensuring safety [52]. The course of labor and the functioning of maternity wards in Polish hospitals are situations often discussed in the media, primarily in connection with negative events. Reports prepared by, among others, the Foundation “Rodzić po ludzku” (“Give birth in a human way”) indicate, for example, that over 50% of women during childbirth or hospitalization in the maternity ward experience some form of abuse by medical personnel [53]. Therefore, many women feel stressed due to the awareness that they will not be accompanied by a partner during childbirth. Women fear loneliness and lack of support, which makes them feel more stressed and anxious about childbirth [36]. Many studies have shown that fear of childbirth decreases if a woman receives support through the presence of a loved one during childbirth. Support during childbirth can shorten its course, is associated with fewer complications and better well-being of women, and a lower risk of postpartum depression or PTSD [7,54,55].

The regression analysis carried out at further stages of the study showed that the predictors of fear of childbirth are stress related to the organization of childbirth and hardiness. These variables explain 29% of the variance of the dependent variable fear of childbirth. Of these, stress related to the organization of childbirth is the most important.

Fear of childbirth occurs in most pregnant women; it can have important consequences, both for the course of childbirth and for the health of the mother and baby. During the COVID-19 pandemic, in many countries, including Poland, the standards of perinatal care changed. Access to doctors was hindered. Prenatal care services were postponed except for mandatory situations, while in some countries, women did not receive gynecological and obstetric care and were only asked to come for labor when it began [55]. Many visits have been limited to teleconsultations. In many countries, women had to attend all visits without a companion, and in some countries (including Poland), family births were banned at the time of the highest number of illnesses, despite the fact that support during labor has a beneficial effect on its course [7,56]. Research suggests that the lack of control over decisions related to labor can be traumatic. Restrictions on hospital visits during and after labor can also be difficult. Women feel that their expectations about labor and prenatal and postpartum care are disrupted, which causes changes to birth plans. In addition, during the pandemic, the number of obstetric staff may be lower than usual due to the transfer of staff to other departments or to infectious diseases hospitals. It may also be important for staff to limit contact with patients for their own safety [56]. The lack of support from a loved one may be particularly difficult for women preparing for childbirth. Studies have shown that support from loved ones is a mediator of fear of childbirth, while during the COVID-19 pandemic, in many countries, women were deprived of this direct support.

It has also been shown that hardiness is a predictor of fear of childbirth. Some authors [57,58] suggest that hardiness may affect the level of anxiety during the pandemic through the assessment of stressful situations. People with a high level of hardiness are aware that changes and negative events are an expected part of life and treat them as challenges that must be faced and dealt with. It is also associated with the use of active coping strategies and more effective adaptation to the new reality. Studies that initiated the concept of hardiness [59,60,61] have shown that people with a high level of hardiness are protected from the negative impact of stress on their functioning, life, and health, including mental health. Many other studies have shown that hardiness is a trait that influences effective coping with difficulties [62]. Studies have shown a negative correlation between hardiness and anxiety [63]. It has also been shown that people with a high level of hardiness are at a lower risk of developing psychopathological symptoms. Studies conducted during the pandemic have shown that hardiness can protect a person from negative emotions [64]. Hardiness allows for the control of anxiety and negative thoughts [22].

Studies have also shown that hardiness is a personal adaptive resource in stressful situations related to the COVID-19 pandemic. Researchers have observed the contribution of the hardness index to the dynamics of anxiety over time. People with lower hardiness showed increasing anxiety over time, while people with medium and high levels of hardiness did not show a significant increase in anxiety levels during the pandemic [42]. On this basis, it can be assumed that hardiness as an personal resource will also affect the level of fear of childbirth. Ref. [18] considers hardiness to be a path that leads a person to resilience and gives courage in uncertainty. In their opinion, people with a high level of hardiness do not feel lost in a difficult situation and remain active. It can therefore be assumed that women with a high level of this feature believe in the capabilities of their bodies, feel that they have control over them, and feel that they will cope, even if they have to give birth in more difficult conditions than before the pandemic. Hardiness can affect women’s belief that their delivery will be successful, which can also reduce anxiety.

In the study, we also showed that the analyzed variables explain only a small percentage of the variance in life satisfaction. Of these, only fear of childbirth was significant. This means that women who feel fear and anxiety related to preparing for childbirth feel less satisfied with life. In a Polish study assessing the level of life satisfaction in pregnant women, it was shown that obstetric variables did not differentiate the level of life satisfaction of the examined women, while social support was very important [65]. The support was limited during the pandemic. In another study, we showed a relationship between fear of childbirth and life satisfaction and that it was a mediator of the relationship between stress and life satisfaction [34]. Other studies have shown that fear of childbirth has many negative consequences for the physical health and life satisfaction of pregnant women. It can disrupt the joy of pregnancy and negatively affect the course of labor [12,66]. Due to hormonal and physiological changes, pregnant women are more sensitive to changes in their environment and more prone to emotional problems [67]. Therefore, fear may have a negative association with women’s life satisfaction [31,68,69,70,71]. However, it should be emphasized that fear of childbirth explains only a small percentage of the variance in life satisfaction of pregnant women during the COVID-19 pandemic.

In summary, most of the analyzed variables were correlated. It was shown that the greater the fear of COVID-19 and the stress experienced by women, the greater the intensity of fear of childbirth. The higher the level of hardiness, the lower the fear of childbirth and the greater the satisfaction with life, and that the higher the fear of childbirth, the lower the satisfaction with life. It was also shown that with age, the fear of COVID-19, hardiness, and satisfaction with life increase, and the younger the person, the higher the level of stress associated with the organization of childbirth. Women planning a family birth experience greater stress associated with the organization of childbirth and fear of childbirth compared to women who do not plan a family birth. The predictors of fear of childbirth are stress associated with the organization of childbirth and hardiness, while the predictor of satisfaction with life is fear of childbirth.

## 5. Limitations

This study is not free from limitations. Longitudinal studies should be conducted to verify the relationships studied. This study was conducted using an online survey during the COVID-19 pandemic. While this allowed for safe and timely data collection, it may have influenced participants’ responses and limited the representativeness of the sample. In addition, during different stages of the pandemic, healthcare systems, including maternity wards, functioned differently, which could be related to the stress and anxiety experienced by women. Maternity care was also organized differently in different countries, which is why it is difficult to transfer the results obtained to the situation in other countries. A limitation of the current study is that although the role of social support is discussed, it was not directly measured. Future research should include validated instruments assessing partner and family support to examine their impact on stress, fear of childbirth, and life satisfaction during pregnancy.

## 6. Conclusions

It can be concluded that epidemics of infectious diseases can have a negative emotional impact on pregnant women, causing fear and stress and contributing to an increased level of fear of childbirth. It should be noted that strong negative emotions that appear during pregnancy can lead to gynecological and obstetric complications and negatively affect the health of the mother and child. Therefore, it is particularly important to support women preparing for childbirth during subsequent pandemics or an increased level of infectious diseases, e.g., during the flu season, and to allow them to give birth in the presence of a companion, which can help reduce the level of stress and anxiety about childbirth. This study showed that hardiness is a significant predictor of fear of childbirth, which is why it is also important to develop personal resources that can have a positive effect on adaptation to a difficult situation, reduce fear of childbirth, and increase life satisfaction. In further studies, it is worth analyzing the role of other personal resources, such as self-efficacy or a sense of coherence, and the role of social support. All the obtained results may be helpful for healthcare workers and, above all, for gynecologists, midwives, and clinical psychologists who provide support to women preparing for childbirth or hospitalized in pregnancy pathology wards.

## Figures and Tables

**Table 1:** Sociodemographic data, variables related to the course of the epidemic, functioning of healthcare and women’s health status.

	M	SD	Min	Max
Age	27.17	4.21	18	39
			n	%
Place of residence	Village		85	34.41%
	City with less than 100.000 inhabitants		71	28.74%
	City of more than 100.000 inhabitants		91	36.84%
Number of pregnancies	First		165	66.80%
	Second		45	18.22%
	Third		27	10.93%
	Fourth		6	2.43%
	Fifth or more		4	1.62%
Number of births	1		185	74.90%
	2		40	16.19%
	3		18	7.29%
	4		3	1.21%
	5 or more		1	0.40%
Problems in anterior pregnancies	Yes		36	14.57%
	No		211	85.43%
Complications during previous births	Yes		13	5.26%
	No		234	94.74%
Chronic diseases	Yes		61	24.70%
	No		186	75.30%
Fear of hospitalization in pregnancy pathology	Yes		150	60.73%
	No		97	39.27%
Planned family birth	Yes		183	74.09%
	No		64	25.91%
Fear of not having an attendant at the birth	Yes		198	80.16%
	No		49	19.84%
Fear of hospital closure	Yes		194	78.54%
	No		53	21.46%
Fear of COVID-19 infection in the hospital	Yes		150	60.73%
	No		97	39.27%

Note. M = mean; Min = minimum value; Max = maximum value; SD = standard deviation.

**Table 2:** Descriptive statistics for the studied variables.

Variable	N	M	Min	Max	SD	SKE	K	W	*p*
Fear of COVID-19	247	19.87	6	30	5.99	−0.25	−0.58	0.98	<0.001
Perinatal infection stress	247	15.82	5	25	5.65	−0.45	−0.87	0.94	<0.001
Preparedness stress	247	25.38	7	35	6.70	−0.75	−0.03	0.94	<0.001
Positive appraisal	247	8.34	3	15	3.21	0.10	−0.82	0.97	<0.001
Fear of childbirth	247	24.99	9	35	5.37	−0.51	0.06	0.98	<0.001
Hardiness	247	49.48	17	71	8.90	−0.24	0.06	0.99	0.190
Life satisfaction	247	23.52	5	35	6.09	−0.64	0.25	0.97	<0.001

Note. N = sample size; M = mean; Min = minimum value; Max = maximum value; SD = standard deviation; SKE = skewness; K = kurtosis; W = Shapiro–Wilk test statistic; *p* = significance level for Shapiro–Wilk test.

**Table 3:** The relationship between fear of COVID-19, stress, fear of childbirth, hardiness, and life satisfaction in the group of pregnant women.

Variable	Fear of COVID-19	Perinatal Infection Stress	Preparedness Stress	Positive Appraisal	Fear of Childbirth	Hardiness	Life Satisfaction
Fear of COVID-19		0.74 ***	0.51 ***	0.29 ***	0.32 ***	−0.07	0.02
Perinatal infection stress	0.74 ***		0.53 ***	0.31 ***	0.33 ***	−0.05	0.06
Preparedness stress	0.51 ***	0.53 ***		0.22 **	0.47 ***	−0.14 *	−0.03
Positive appraisal	0.29 ***	0.31 ***	0.22 **		−0.08	−0.07	0.19 **
Fear of childbirth	0.32 ***	0.33 ***	0.47 ***	−0.08		−0.22 ***	−0.29 ***
Hardiness	−0.07	−0.05	−0.14 *	−0.07	−0.22 ***		0.15 *
Life satisfaction	0.02	0.06	−0.03	0.19 **	−0.29 ***	0.15 *	

*** *p* < 0.001; ** *p* < 0.01; * *p* < 0.05.

**Table 4:** Sociodemographic variables and the level of fear of COVID-19, stress, fear of childbirth, hardiness, and life satisfaction.

Variable	Age	Number of Pregnancies	Number of Births	Week of Pregnancy
Fear of COVID-19	0.16 *	0.04	0.03	0.04
Perinatal infection stress	0.09	0.05	0.04	−0.01
Preparedness stress	−0.13 *	−0.15 *	−0.22 ***	0.03
Positive appraisal	0.01	−0.01	−0.03	−0.01
Fear of childbirth	−0.10	−0.22 ***	−0.27 ***	−0.02
Hardiness	0.16 *	−0.05	0.02	0.13 *
Life satisfaction	0.15 *	−0.03	−0.09	0.04

* *p* < 0.05; *** *p* < 0.001.

**Table 5:** Planned family birth and the level of analyzed variables.

Variable	Yes (N = 183)		No (N = 64)		t	df	*p*
	M	SD	M	SD			
Fear of COVID-19	20.20	5.99	18.94	5.93	1.46	245	0.15
Perinatal infection stress	16.07	5.56	15.11	5.90	1.17	245	0.24
Preparedness stress	26.73	6.09	21.52	6.91	5.69	245	<0.001
Positive appraisal	8.42	3.23	8.13	3.18	0.62	245	0.54
Fear of childbirth	2.40	5.26	23.84	5.56	2.01	245	<0.05
Hardiness	49.26	8.48	50.13	10.04	−0.67	245	0.50
Life satisfaction	23.68	5.95	23.05	6.48	0.72	245	0.47

Note. N = sample size; M = mean; SD = standard deviation; t = *t*-test value; df = degrees of freedom; *p* = significance level.

**Table 6:** Predictors of fear of childbirth.

Predictors	B	SE	Beta	t	*p*	Model Summary		
						F _(4,242)_	*p*	R^2^
Fear of COVID-19	0.06	0.08	0.06	0.76	0.45			
Perinatal infection stress	0.07	0.08	0.07	0.82	0.41			
Preparedness stress	0.30	0.05	0.38	5.60	<0.001	20.8	<0.001	0.24
Hardiness	−0.10	0.03	−0.16	−2.84	<0.001			

Note. B = unstandardized regression coefficient; SE = standard error; Beta = standardized regression coefficient; t = *t*-test value; *p* = significance level; R^2^ = coefficient of determination; F = F-test value for overall model significance.

**Table 7:** Predictors of life satisfaction.

Predictors	B	SE	Beta	t	*p*	Model Summary		
						F _(6,240)_	*p*	R^2^
Fear of COVID-19	−0.02	0.09	−0.02	−0.20	0.84			
Perinatal infection stress	0.12	0.10	0.11	1.20	0.23			
Preparedness stress	0.04	0.07	0.05	0.62	0.53			
Positive appraisal	0.24	0.12	0.13	1.94	0.05	6.13	<0.05	0.13
Fear of childbirth	−0.35	0.08	−0.31	−4.28	<0.001			
Hardiness	0.07	0.04	0.10	1.67	0.10			

Note. B = unstandardized regression coefficient; SE = standard error; Beta = standardized regression coefficient; t = *t*-test value; *p* = significance level; R^2^ = coefficient of determination; F = F-test value for overall model significance.

## Data Availability

The data can be made available from the corresponding author upon reasonable request.

## References

[1] Masmejan S., Pomar L., Lepigeon K., Favre G., Baud D., Rieder W. (2020). COVID-19 and pregnancy. Rev. Médicale Suisse.

[2] Wang C.L., Liu Y.Y., Wu C.H., Wang C.Y., Wang C.H., Long C.Y. (2021). Impact of COVID-19 on Pregnancy. Int. J. Med. Sci..

[3] Chaudhry S., Aboudawoud O., Hardy G. (2023). A History of COVID-19 in Pregnancy: A Narrative Review. JCM.

[4] Chen H., Guo J., Wang C., Luo F., Yu X., Zhang W., Li J., Zhao D., Xu D., Gong Q. (2020). Clinical characteristics and intrauterine vertical transmission potential of COVID-19 infection in nine pregnant women: A retrospective review of medical records. Lancet.

[5] Zambrano L.D., Ellington S., Strid P., Galang R.R., Oduyebo T., Tong V.T., Woodworth K.R., Nahabedian J.F., Azziz-Baumgartner E., Gilboa S.M. (2020). Update: Characteristics of Symptomatic Women of Reproductive Age with Laboratory-Confirmed SARS-CoV-2 Infection by Pregnancy Status—United States, January 22–October 3, 2020. MMWR Morb. Mortal. Wkly. Rep..

[6] Giesbrecht G.F., Rojas L., Patel S., Kuret V., MacKinnon A.L., Tomfohr-Madsen L., Lebel C. (2022). Fear of COVID-19, mental health, and pregnancy outcomes in the pregnancy during the COVID-19 pandemic study. J. Affect. Disord..

[7] Dymecka J., Gerymski R., Iszczuk A., Bidzan M. (2021). Fear of Coronavirus, Stress and Fear of Childbirth in Polish Pregnant Women during the COVID-19 Pandemic. Int. J. Environ. Res. Public Health.

[8] Preis H., Mahaffey B., Heiselman C., Lobel M. (2020). Vulnerability and resilience to pandemic-related stress among U.S. women pregnant at the start of the COVID-19 pandemic. Soc. Sci. Med..

[9] Ilska M., Kołodziej-Zaleska A., Brandt-Salmeri A., Preis H., Lobel M. (2021). Pandemic-related pregnancy stress assessment–Psychometric properties of the Polish PREPS and its relationship with childbirth fear. Midwifery.

[10] Larkin P., Begley C.M., Devane D. (2009). Women’s experiences of labour and birth: An evolutionary concept analysis. Midwifery.

[11] Geissbuehler V., Eberhard J. (2002). Fear of childbirth during pregnancy: A study of more than 8000 pregnant women. J. Psychosom. Obstet. Gynecol..

[12] Nilsson C., Hessman E., Sjöblom H., Dencker A., Jangsten E., Mollberg M., Patel H., Sparud-Lundin C., Wigert H., Begley C. (2018). Definitions, measurements and prevalence of fear of childbirth: A systematic review. BMC Pregnancy Childbirth.

[13] Saris W.E., Veenhoven R., Scherpenzeel A.C., Bunting B. (1996). The study of life—Satisfaction. A Comparative Study of Satisfaction with Life in Europe.

[14] Diener E. (1984). Subjective well-being. Psychol. Bull..

[15] Pavot W., Diener E. (2008). The Satisfaction with Life Scale and the emerging construct of life satisfaction. J. Posit. Psychol..

[16] Dymecka J., Gerymski R., Machnik-Czerwik A. (2020). Fear of COVID-19 as a buffer in the relationship between perceived stress and life satisfaction in the Polish population at the beginning of the global pandemic. Health Psychol. Rep..

[17] Dymecka J., Gerymski R., Machnik-Czerwik A. (2022). How does stress affect life satisfaction during the COVID-19 pandemic? Moderated mediation analysis of sense of coherence and fear of coronavirus. Psychol. Health Med..

[18] Maddi S.R. (2007). Relevance of Hardiness Assessment and Training to the Military Context. Mil. Psychol..

[19] Dymecka J., Bidzan-Bluma I., Bidzan M., Borucka-Kotwica A., Atroszko P., Bidzan M. (2020). Validity and reliability of the Polish adaptation of the Health-Related Hardiness Scale—The first confirmatory factor analysis results for a commonly used scale. Health Psychol. Rep..

[20] Sunderlin C.M. (2007). The Relationship Between Risk, Hardiness and Social Support on Perceived Quality of Life Among Individuals Diagnosed with Fibromyalgia. Ph.D. Thesis.

[21] Wilder B.F. (1995). The Relationship of Selected Demographic Characteristics, Health Conditions, and Personality Hardiness with Quality of Life in Older Adults. Ph.D. Thesis.

[22] Buheji M., Jahrami H. (2020). Analysing Hardiness Resilience in COVID-19 Pandemic—Using Factor Analysis. Int. J. Manag..

[23] Vagni M., Maiorano T., Giostra V., Pajardi D. (2020). Hardiness, Stress and Secondary Trauma in Italian Healthcare and Emergency Workers during the COVID-19 Pandemic. Sustainability.

[24] Putyński L., Paciorek M. (2008). Kwestionariusz Lęku Porodowego (KLP II) Wersja Zrewidowana—Konstrukcja i właściwości psychometryczne. Acta Univ. Lodz. Folia Psychol..

[25] Pollock S.E. (1986). Human responses to chronic illness: Physiologic and psychosocial adaptation. Nurs. Res..

[26] Czapiński J. (2004). Psychologia Pozytywna. Nauka o Szczęściu, Zdrowiu, Sile i Cnotach Człowieka.

[27] Lumley T., Diehr P., Emerson S., Chen L. (2002). The importance of the normality assumption in large public health data sets. Stat. Med..

[28] Blanca M.J., Alarcón R., Arnau J., Bono R., Bendayan R. (2017). Non-normal data: Is ANOVA still a valid option?. Psychology.

[29] Hill T., Lewicki P. (2007). Statistics: Methods and Applications.

[30] Bjelica A. (2004). Pregnancy as a stressful life event and strategies for coping with stress in women with pregnancy-induced hypertension. Med. Pregl..

[31] Dymecka J., Filipkowski J., Machnik-Czerwik A. (2021). Fear of COVID-19: Stress and job satisfaction among Polish doctors during the pandemic. Adv. Psychiatry Neurol..

[32] Satici B., Gocet-Tekin E., Deniz M.E., Satici S.A. (2021). Adaptation of the Fear of COVID-19 Scale: Its Association with Psychological Distress and Life Satisfaction in Turkey. Int. J. Ment. Health Addict..

[33] Ahorsu D.K., Lin C.Y., Imani V., Saffari M., Griffiths M.D., Pakpour A.H. (2022). The Fear of COVID-19 Scale: Development and Initial Validation. Int. J. Ment. Health Addict..

[34] Gerymski R., Dymecka J., Iszczuk A., Bidzan M. (2023). Perceived stress and life satisfaction in pregnant women during the COVID-19 pandemic: The mediating role of fear of childbirth and self-esteem. Health Psychol. Rep..

[35] Zhang Y., Ma Z.F. (2020). Impact of the COVID-19 Pandemic on Mental Health and Quality of Life among Local Residents in Liaoning Province, China: A Cross-Sectional Study. Int. J. Environ. Res. Public Health.

[36] Studniczek A., Kossakowska K. (2020). Ciąża i poród w czasach pandemii COVID-19: Wybrane aspekty psychologiczne. Kwart. Nauk. Fides Ratio.

[37] Farber E.W., Schwartz J.A.J., Schaper P.E., Moonen D.J., McDaniel J.S. (2000). Resilience Factors Associated with Adaptation to HIV Disease. Psychosomatics.

[38] Strycharczyk D., Clough P. (2024). ODPORNOŚĆ PSYCHICZNA Strategie i Narzędzia Rozwoju.

[39] Dymecka J., Gerymski R., Machnik-Czerwik A., Derbis R., Bidzan M. (2021). Fear of COVID-19 and Life Satisfaction: The Role of the Health-Related Hardiness and Sense of Coherence. Front. Psychiatry.

[40] Manning M.R., Williams R.F., Wolfe D.M. (1988). Hardiness and the relationship between stressors and outcomes. Work Stress.

[41] Kobasa S.C. (1979). Stressful life events, personality, and health: An inquiry into hardiness. J. Personal. Soc. Psychol..

[42] Epishin V.E., Salikhova A.B., Bogacheva N.V., Bogdanova M.D., Kiseleva M.G. (2020). Mental Health and the COVID-19 Pandemic: Hardiness and Meaningfulness Reduce Negative E!ects on Psychological Well-Being. Psychol. Russ..

[43] Aubi S., Teimory S., Nayyeri M. (2010). Hardiness, quality of life and well-being. Dev. Psychol. J. Iran. Psychol..

[44] Maddi S.R., Kahn S., Maddi K.L. (1998). The effectiveness of hardiness training. Consult. Psychol. J. Pract. Res..

[45] Bielawska-Batorowicz E. (2006). Psychologiczne Aspekty Prokreacji.

[46] Delbaere I., Verstraelen H., Goetgeluk S., Martens G., De Backer G., Temmerman M. (2007). Pregnancy outcome in primiparae of advanced maternal age. Eur. J. Obstet. Gynecol. Reprod. Biol..

[47] Kubiak-Fortecka A., Wilczyński J. (2009). Pregnancy and delivery in women over 35 years old. Menopause Rev./Przegląd Menopauzalny.

[48] Łuczak-Wawrzyniak J., Czarnecka-Iwańczuk M., Bukowska A., Konofalska N. (2010). Early and late psychological effects of pregnancy loss. Ginekol. Pol..

[49] Kicia M., Skurzak A., Wiktor K., Iwanowicz-Palus G., Wiktor H. (2015). Anxiety and stress in miscarriage. Pol. J. Public Health.

[50] Kamasz E., Pilarska N., Włodarczyk A. (2020). Znaczenie traumatycznych narracji porodowych oraz oceny opieki ginekologicznej jako czynnika rozwoju tokofobii w oczach młodych kobiet. Kwart. Nauk. Fides Ratio.

[51] Kelner M. (1988). Unraveling the Mystery of Health: How People Manage Stress and Stay Well. Aaron Antonovsky. Jossey-Bass Publishers, San Francisco, 1987. $35.00. Can. J. Aging.

[52] Stadnicka G., Pawlowska-Muc A. (2015). Poród rodzinny w opinii kobiet i ich współmałżonków/partnerów. Rocz. Teol..

[53] (2021). Raport “Opieka Okołoporodowa Podczas Pandemii COVID-19 w Świetle Doświadczeń Kobiet i Personelu Medycznego”. Fundacja Rodzić po Ludzku. https://rodzicpoludzku.pl/raporty/raport-opieka-okoloporodowa-podczas-pandemii-covid-19-w-swietle-doswiadczen-kobiet-i-personelu-medycznego/.

[54] Świątkowska-Freund M., Kawiak D., Preis K. (2007). Advantages of father’s assistance at the delivery. Ginekol. Pol..

[55] Mizrak Sahin B., Kabakci E.N. (2021). The experiences of pregnant women during the COVID-19 pandemic in Turkey: A qualitative study. Women Birth.

[56] Brooks S.K., Weston D., Greenberg N. (2020). Psychological impact of infectious disease outbreaks on pregnant women: Rapid evidence review. Public Health.

[57] Fisher C., Hauck Y., Fenwick J. (2006). How social context impacts on women’s fears of childbirth: A Western Australian example. Soc. Sci. Med..

[58] Bartone P.T., McDonald K., Hansma B.J., Solomon J. (2022). Hardiness moderates the effects of COVID-19 stress on anxiety and depression. J. Affect. Disord..

[59] Kobasa S.C., Maddi S.R., Courington S. (1981). Personality and Constitution as Mediators in the Stress-Illness Relationship. J. Health Soc. Behav..

[60] Kobasa S.C., Maddi S.R., Kahn S. (1982). Hardiness and health: A prospective study. J. Personal. Soc. Psychol..

[61] Kobasa S.C., Maddi S.R., Puccetti M.C. (1982). Personality and exercise as buffers in the stress-illness relationship. J. Behav. Med..

[62] Jankowska M. (2010). Hardiness jako narzędzie wspomagające radzenie sobie w sytuacji kryzysowej—Przegląd badań. Cent. Eur. J. Soc. Sci..

[63] Abdollahi A., Abu Talib M., Carlbring P., Harvey R., Yaacob S.N., Ismail Z. (2018). Problem-solving skills and perceived stress among undergraduate students: The moderating role of hardiness. J. Health Psychol..

[64] Vagni M., Maiorano T., Giostra V., Pajardi D. (2020). Coping with COVID-19: Emergency Stress, Secondary Trauma and Self-Efficacy in Healthcare and Emergency Workers in Italy. Front. Psychol..

[65] Kaźmierczak M., Gebuza G., Topolińska M., Gierszewska M., Mieczkowska E. (2018). Ocena stopnia satysfakcji z życia u kobiet w ciąży. Pielegniarstwo Pol..

[66] Ryding E.L., Wijma K., Wijma B. (1998). Psychological impact of emergency Cesarean section in comparison with elective Cesarean section, instrumental and normal vaginal delivery. J. Psychosom. Obstet. Gynecol..

[67] Heron J., O’Connor T.G., Evans J., Golding J., Glover V. (2004). The course of anxiety and depression through pregnancy and the postpartum in a community sample. J. Affect. Disord..

[68] Pazzagli C., Laghezza L., Capurso M., Sommella C., Lelli F., Mazzeschi C. (2015). ANTECEDENTS AND CONSEQUENCES OF FEAR OF CHILDBIRTH IN NULLIPAROUS AND PAROUS WOMEN: Fear of Childbirth in Nulliparous and Parous Women. Infant Ment. Health J..

[69] Saisto T., Halmesmäki E. (2003). Fear of childbirth: A neglected dilemma. Acta Obstet. Gynecol. Scand..

[70] Webster J., Nicholas C., Velacott C., Cridland N., Fawcett L. (2011). Quality of life and depression following childbirth: Impact of social support. Midwifery.

[71] Yu M., Qiu T., Liu C., Cui Q., Wu H. (2020). The mediating role of perceived social support between anxiety symptoms and life satisfaction in pregnant women: A cross-sectional study. Health Qual. Life Outcomes.

